# Enhancing the Potentiality of *Trichoderma harzianum* against *Pythium* Pathogen of Beans Using Chamomile (*Matricaria chamomilla*, L.) Flower Extract

**DOI:** 10.3390/molecules26041178

**Published:** 2021-02-22

**Authors:** Abeer Abdulkhalek Ghoniem, Kamar M. Abd El-Hai, Ayman Y. El-khateeb, Noha M. Eldadamony, Samy F. Mahmoud, Ashraf Elsayed

**Affiliations:** 1 Microbial Activity Unit, Department of Microbiology, Soils, Water and Environment Research Institute, Agricultural Research Center, Giza 12619, Egypt; abeer.abdelkhalik@yahoo.com; 2 Department of Leguminous and Forage Crop Diseases, Plant Pathology Research Institute, Agricultural Research Center, Giza 12112, Egypt; Kamar_1968@yahoo.com; 3 Department of Agricultural Chemistry, Faculty of Agriculture, Mansoura University, Elgomhouria St., Mansoura 35516, Egypt; aymanco@mans.edu.eg; 4 Seed Pathology Department, Plant Pathology Institute, Agricultural Research Center, Giza 12112, Egypt; Nohamohamadt@gmail.com; 5 Department of Biotechnology, College of Science, Taif University, P.O. Box 11099, Taif 21944, Saudi Arabia; s.farouk@tu.edu.sa; 6 Botany Department, Faculty of Science, Mansoura University, Elgomhouria St., Mansoura 35516, Egypt

**Keywords:** *Trichoderma harzianum*, antifungal activity, *Matricaria chamomilla*, *Phaseolus vulgaris*

## Abstract

Our present study was designed to investigate the role of both *Trichoderma harzianum* and chamomile (*Matricaria chamomilla* L.) flower extract in mutual reaction against growth of *Pythium ultimum*. In vitro, the activity of chamomile extract was found to reduce the radial growth of *Pythium ultimum* up to 30% compared to the control. Whereas, the radial growth reduction effect of *T. harzianum* against *P. ultimum* reached 81.6% after 120 h. Data also showed the productivity of total phenolics and total flavonoids by *T. harzianum*, was 12.18 and 6.33 mg QE/100 mL culture filtrate, respectively. However, these compounds were determined in chamomile flower extract at concentrations of 75.33 and 24.29 mg QE/100 mL, respectively. The fractionation of aqueous extract of chamomile flower using HPLC provided several polyphenolic compounds such as pyrogallol, myricetin, rosemarinic acid, catechol, *p*-coumaric acid, benzoic acid, chlorogenic acid and other minor compounds. In vivo, the potentiality of *T. harzianum* with chamomile flower extract against *Pythium* pathogen of bean was investigated. Data obtained showed a reduction in the percentage of rotted seed and infected seedling up to 28 and 8%, respectively. Whereas, the survival increased up to 64% compared to other ones. There was also a significant promotion in growth features, total chlorophyll, carotenoids, total polyphenols and flavonoids, polyphenol-oxidase and peroxidase enzymes compared to other ones. To the best of our knowledge, there are no reported studies that included the mutual association of fungus, *T. harzianum* with the extract taken from the chamomile flower against *P. ultimum*, either in vitro or in vivo. In conclusion, the application of both *T. harzianum* and/or *M. chamomilla* extracts in the control of bean *Pythium* pathogen showed significant results.

## 1. Introduction

Leguminous crops, such as *Phaseolus vulgaris*, L. are widely cultivated for human consumption, due to their richness in protein, fibers, calories, vitamin B and other minerals, e.g., iron, calcium, phosphorous and zinc [1,2]. Common French beans are a variant of those legume crops that contain several constituents, i.e., flavonoids, saponins, tannins and phenolic acid, with significant biological activity, e.g., anti-urolithiatic and anti-obesity [3]. However, these bean plants often get infected by pathogenic species of *Pythium* spp., which cause decay, pre-emergency and post-emergency on bean species. Additionally, the infected common bean seeds or seedlings turned to be discolored, chlorotic and soft and decay after germination and/or they become wilt or die within 1–3 weeks [4].

Several pathogens are known to infect been plants and have a significant economic impact on the bean market. The most aggressive species of *Pythium*, *P. aphanidermatum*, affects bean plants causing root rot and crown necrosis of mature bean plants, which has a significant economic impact [5,6]. Another species is *P*. *splendens*, which is known for its pathogenicity towards seedlings of different species, causing damping-off of seedlings [7]. The most common *Rhizoctonia solani*, *Fusarium solani*, *F. oxysporum* and *Pythium ultimum* had superiority among the soil borne fungi in causing pathogens of beans [8]. Importantly, several investigations pointed out that the colored seeds of beans cultivars were more resistant to *Pythium pathogens* compared to other white seeds [9]. Therefore the choice in procedures of pathogen control, which have differing environmental impact, depends on the type of the bean cultivars and their resistance which has an effect on sustaining the agricultural process.

Sustaining the agricultural process with a high crop production, requires protection strategies of which disease control plays a crucial role in the management and protection against plant diseases which cause severe loss of crop during epidemics [10]. Fungicides and pesticides are commonly used to combat fungal and insect diseases. However, their active chemical agents often have deleterious effects on the environment so the search for environmentally friendly alternatives has become significant.

The biological control of these diseases using *Trichoderma* spp. has been gaining interest worldwide [11], due to the reported efficacy and potential of *Trichoderma* spp against soil borne fungi [12]. Members of the *Trichoderma* genus of fungi belong to the family Hypocreaceae, which is present in nearly all soil types. They are the most prevalent culturable fungi and can be characterized as opportunistic avirulent plant symbionts that can form mutualistic endophytic relationships with several plant species [13]. The therapeutic bioagent, *Trichoderma* spp. employs numerous mechanisms involved in the restriction of other fungi including enhanced promotion of plants through the vegetation period of growth. Examples of such mechanisms involved include mycoparasitism, antibiosis activity, competition, chitinase and gluconase enzymes production [14,15] additionally, the defense responses with metabolism of germination stimulants and promotion of systemic acquired resistance [10,16,17]. Other studies showed that *Trichoderma* spp are able to secrete several biologically active agents of significance, e.g., pyrones, isocyanates, peptides, peptaibols and trichothenes [18]. *Trichoderma* spp. are promising new therapeutic agents as they are potentially safer and effective than commercially available alternatives in addition to being more environmentally safer posing relatively no hazardous effect to food chains [19].

Plant extracts are considered another alternative to traditional fungicides and pesticides as they produce bioagents that are effective against bacteria and fungi [20]. The volatile compounds and essential oils show promise as a substitute for antibacterial and antifungal agents in several studies [21]. Additionally, increasing attention has been directed towards extracts of grapefruit and citrus wastes which contain bioactive molecules that have anti-fungal properties as well as the ability to further induce plant resistance [22,23]. Similarly, the biological activity of *Capparis spinosa* has been investigated, by which the scavenging activity against DPPH and ABTS radical cations could be due to the high content of phenolic compounds such as flavonoids and tannins [24]. Najjaa, et al. [25] investigated the high content of polyphenol (164.85 GEA/g extract) of herbal plant, i.e., *Retama reatam*, which has antimicrobial properties. Chamomile is one of the most consumed herbal teas in the world extracted from chamomile (*Matricaria chamomilla*, L.) which is used in therapeutic spectrums, e.g., anti-inflammatory, analgesic, antimicrobial, antispasmic and sedative [26]. The therapeutic use of the chamomile is due to it being an excellent source of bioactive molecules such as phenolic, terpenoids, essential oil and flavonoids content [27,28]. Moreover, the high content of phenolic compounds, especially the subfamily of flavonoids are the most responsible for its high antioxidant activity which can be utilized through extraction from chamomile plants [29].

The extraction strategies for phytonutrients of chamomile could be subjected to several techniques, of which several employ the use of environmentally safe and nontoxic solvents [30]. In general, the extraction of various phytochemicals, specifically polyphenolic compounds is effectively done by solvents such as methanol, ethanol, glycerol and water [31]. However, more and more attention has been focused towards the use of water as a safe effective solvent for the extraction of phenolic compounds [32].

This study has been designed to investigate the potentiality of *T. harzianum* towards *P. ultimum*, in association with aqueous extract of chamomile (*Matricaria chamomilla*, L.) flower, either in vitro and/or in vivo.

## 2. Results and Discussion

Generally, the bean is regarded as an important nutritional crop in combating starvation for millions of people worldwide [33,34], but it is prone to diseases throughout its life cycle, especially ones that are caused by soil borne fungi [35]. Fungicide threshold chemical agents are often used to effectively protect plants from soil borne diseases, however their use is restricted due to their immensely harmful effect on the environment leading to the emergence of mutant fungal variations of pathogenic species as well as biomagnification that extends to human food products affecting their health [36,37]. Recently, both biotic and naturally occurring abiotic agents have shown to be the best approaches in perspective study plant disease control, due to their relative safety towards the environment. In this study, *T. harzianum* alone and/or associated with chamomile (*Matricaria chamomilla*, L.) flower extract was investigated against the bean (*Phaseolus vulgaris,* L.) pathogen *P. ultimum*, in vitro and in vivo.

### 2.1. In Vitro, the Comparative Response of P. ultimum to Aqueous Extract of Chamomile Flower and T. harzianum

**Figure 1:**
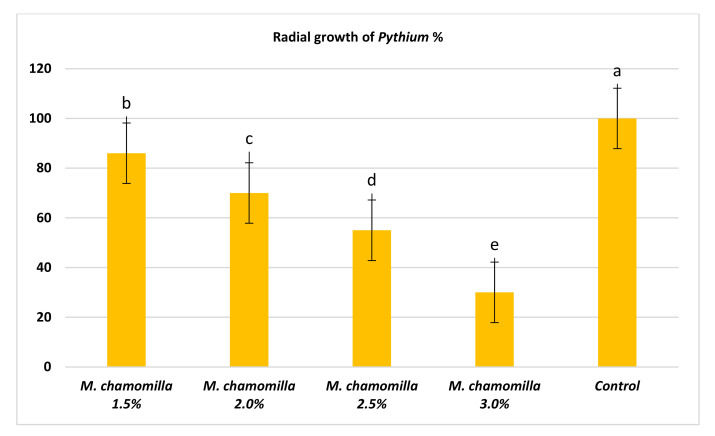
Radial growth (%) of *P*. *ultimum* as a responded to thresholds of chamomile flower extract.

Our study was done to evaluate the potentiality of chamomile flower extract on the radial growth of *P. ultimum*. Data shown in Figure 1 indicates the superiority of chamomile extract at concentrations of 3.0%, compared to other treatments where the radial growth of *P. ultimum* decreased significantly to 30% compared to the control. The radial growth of *P. ultimum* responded significantly to different concentrations of chamomile extract when compared to the control. These results could be due to the total phenolic compounds and total flavonoids, 75.33 and 24.29 mg QE/100 mL filtrate, respectively contained in chamomile.

**Figure 2:**
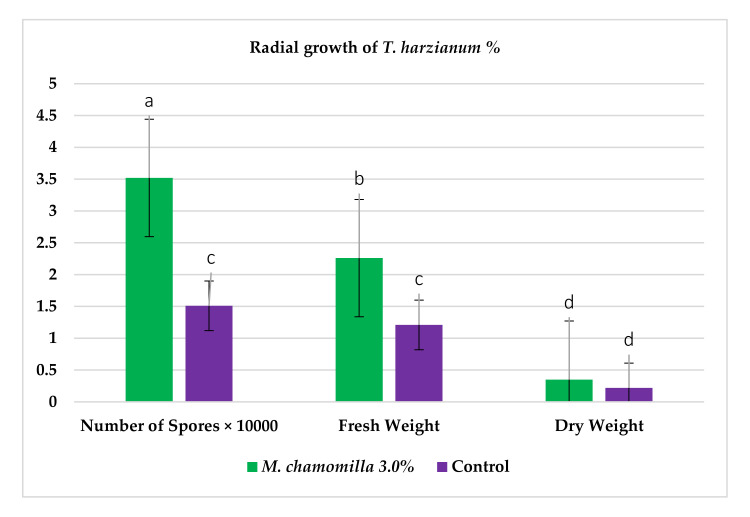
Enhancement of the radial growth of *T. harzianum* as threshold of chamomile flower extract compared to the control.

**Figure 3:**
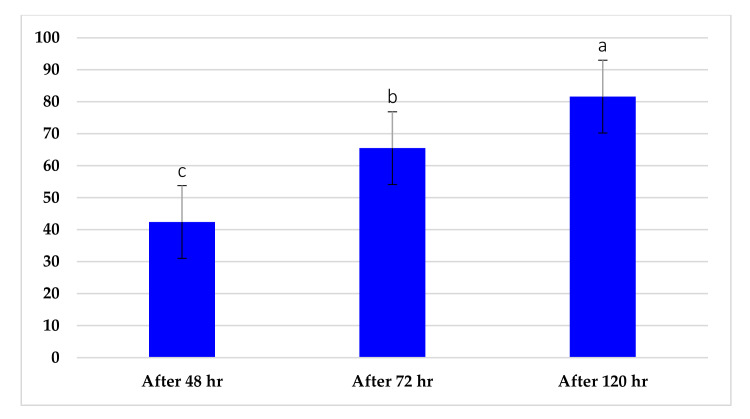
Growth reduction percentage of *P. ultimum* as antibiosis of *T. harzianum*.

Constituents of chamomile flower extract (Table 1), were determined during the fractionation of the aqueous extract of chamomile using high performance liquid chromatography (HPLC showing several common phenolic compounds, such as myricetin (1587.82 ppm), quercetin (927.72 ppm), benzoic acid (414.88 ppm), rosemarinic acid (370 ppm), which were provided as major constituents. Other components showed minor values, e.g., catechol (11.28 ppm), coumaric acid (23.59 ppm) and chlorogenic acid (38.02). These results are consistent with other reported studies [26,28]. Likewise, Raal et al. [27] investigated the essential oil, terpenoids and polyphenols content in commercial chamomile extract. Polyphenolic compounds and their derivatives could be play a crucial role as an alternative to antifungal activity [38]. However, it is interestingly that, the chamomile extract was found to *significantly* sustain the growth features of *T. harzianum*, i.e., the fresh weight and number of spores compared to the control (Figure 2). However, the dry weight showed no significant difference compared to the control. Consequently, the chamomile flower extract was chosen as applicable bioactive agent in association with *T. harzianum* in the management of *Pythium* pathogen in vivo. The antagonistic activity of *T. harzianum* against *P. ultimum* during a dual culture procedure was studied, by which the radial growth of *P. ultimum* decreased significantly with a ratio of 81.6%, after 120 h compared to the control (Figure 3). There is also a significant difference among the interval periods.

The total phenolic compounds and total flavonoids content of culture filtrate of *T. harzianum* were 12.18 and 6.33 mg QE/100 mL culture filtrate, respectively. Furthermore, the kinetic process of how the *Trichoderma* spp. preys on the host pathogen, could be occurred during the mycoparasitic interaction, by secretion of chitinase and β-1,3-glucanase lytic enzymes that attack the host fungi bearing holes in the walls of the fungi and plucking the nutrients which reduces their growth [39]. Moreover, the potentiality of *Bacillus pumilus* INR7, *Trichoderma harzianum* and *Rhizophagus intraradices* against *Rhizoctonia* root rot of common bean (*Phaseolus vulgaris*, L.) has been investigated, in addition to their extended efficiency at the reduction of disease severity and improving dry weight of the bean [40]. The potentiality of *Trichoderma* spp. towards some pathogenic fungi, e.g., *Alternaria alternata, Macrophomina phaseolina* and *Geotrichum candidum* was investigated. T. viride was found to reduce the fungal growth of *A. alternata, M. phaseolina* and *G*. *candidum* up to 84.44, 86.66 and 74.44% respectively [41]. Similarly, the *Rhizobium* sp along with cyanobacterial extracts significantly inhibited the mycelial growth of *Sclerotinia sclerotiorum* that causes the white rot disease of the common bean [42].

### 2.2. In Vivo, Investigation of the Potentiality of Chamomile Flower Extract and Culture of T. Harzianum Against Pythium Pathogen of Bean

#### 2.2.1. Disease Assessment

The percentage of rotted seeds and infected seedlings as well as the survival of *Phaseolus vulgaris* plants as they responded to different treatments were recorded in Table 2 across the board the bean pathogen disease incidence% was decreased significantly in all treatments. a combined mixture of chamomile flower extract and *T. harzianum* showed the most promise and rose to second order after fungicide, with a highly significance increase of rotted seed, infected seedling and survival percentages. However, the fungicide increased the survival percent up to 84%, whereas, both of chamomile extract and *T. harzianum* increased the survival percentage of beans by 58 and 54%, respectively. The survival percentages of beans were significantly responsive to therapeutic treatments, and this response is likely due to the presence of flavonoids and phenolics compounds in the extract and/or associated with growth of fungus, *Trichoderma* spp [38]. Negi et al. [15] investigated the role of *T. viride*, *T. harzianum* and T. virens as bioagents against *Phaseolus vulgaris* diseases, in which the *Trichoderma* spp antagonized many of the plant pathogens, additionally, they served a role as plant growth promoters. The efficiency of plant growth promoting rhizobacteria play a crucial role in protection of *Phaseolus vulgaris* beans against fungal diseases [15]. Similarly, the synergism of cyanobacterial extracts with Rhizobium leguminosarum diminished the disease incidence and severity in common bean (*Phaseolus vulgaris*, L.) during infection with *Sclerotinia sclerotiorum* [42]. The chamomile flower extract was effective but less efficient compared to the mixture of extract with *T. harzianum* in disease parameters. The efficiency of chamomile extract is likely due to the involvement of antioxidant compounds, e.g., flavonoids, coumarins, phenolic acids, glucosides, terpenoids, vitamins and sesquiterpenes [26,27]. The efficacy of *Trichderma harzianum* C52 in reducing the disease percentage and disease incidence of lettuce, was 50 and 35%, respectively [43]. McLean, et al. [44] investigated the role of *Trichoderma harzianum* C52 in controlling the onion white rot pathogen, *Sclerotium cepivorum*.

#### 2.2.2. Morphological Features of Bean Plants

**Figure 4:**
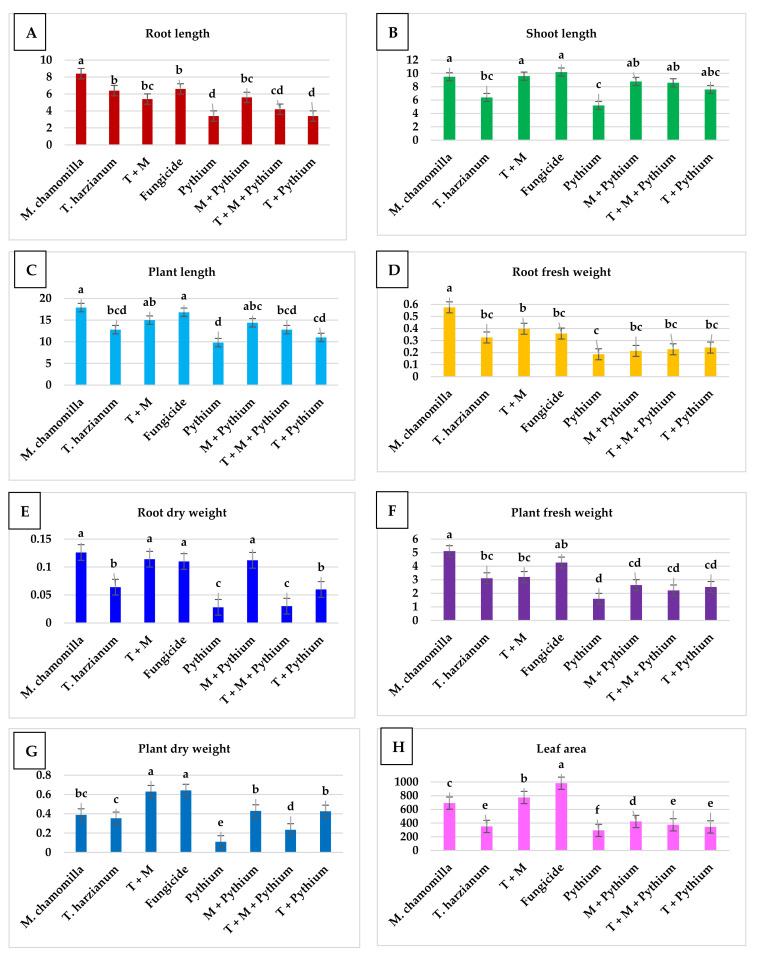
(**A**) root length, (**B**) shoot length, (**C**) plant length (**D**) root fresh weight, (**E**) root dry weight, (**F**) plant fresh weight and (**G**) plant dry weight, as well as, (**H**) leaf area of *Phaseolus vulgaris* as response to different treatments.

Data as depicted in Figure 4 illustrated the values of plant length (root and shoot length), root fresh weight, root dry weight and plant dry weight, as well as, leaf area as the samples responded to different treatments. Concerning plant length of beans, chamomile extract showed a high value of length compared to other treatments, however no significant difference of length values were found with the use of fungicide and a mixture treatment (extract and *T. harzianum*). Furthermore, the chamomile extract showed superiority in root fresh weight, with significant value increases compared to the other treatments with no significant difference among the mixture, fungicide and *T. harzianum* treatments in plant fresh weight. Regarding leaf area, the fungicide showed the highest values followed by the mixture and chamomile, respectively. Similarly, the efficiency of both Bacillus subtilis ATCC11774 with and/or without amino acids mixture against wilt disease pathogen of tomato has been investigated, by which the response in plant growth features, e.g., root and shoot length, shoot fresh and dry weight of tomato plants to Bacillus subtilis with a mixture of amino acids was occupied [45]. As well as, the management of tomato leaf spots caused by the fungus Alternaria tenuissima, by the use of the bioagents Agrileen and salicylic acid were tested [46].

#### 2.2.3. Physiological Characters

- Total phenols, total flavonoids, defense related enzymes and antioxidant capacity

The response of total phenols, flavonoids and defense related enzymes (polyphenol oxidase and peroxidase), as well as, antioxidant capacity (ABTS% inhibition and DPPH% inhibition) to chamomile extract, *T. harzianum* and its combination under non-infested and infested soil with *P. ultimum* are shown in Table 3. Concerning total polyphenols, the mixture treatment showed higher values when compared to other treatments, whereas, both chamomile and *T. harzianum* provided higher values compared to *Pythium* treatment alone. The tricombination (extract+*Tricho*.+ *Pythium*) showed the highest value compared to each individual extract and *Trichoderma* alone. Interestingly, the mixture of extract and *Trichoderma* provided the highest values of total flavonoids significantly higher than other treatments. Additionally, there was an increase in responsiveness shown in values of treatments using polyphenol oxidase and peroxidase compared to *Pythium* alone. Otherwise, the ABTS and DPPH inhibition% were increased significantly in case of a mixture, followed by fungicide, whereas in a case of *Pythium* alone, a less value has been obtained compared to other ones. These data are conceding with the investigation of Ghoniem, et al. [45] who found that an increased response of total polyphenols, total flavonoids, and polyphenoloxidase in tomato plants as result of *Bacillus subtilis* and a mixture of amino acids. Further, *Trichoderma* spp. could be produced effectively plant growth promoters, such as gibberellic acid and biological control of some pathogenic fungi, i.e., *Rhizoctonia solani*, with increasing the plant growth [47,48].

Our results implicated that the content of chamomile extract of phenols and flavonoids, as well as, the productivity of such these a aforementioned compounds by *T. harzianum* could be responsible for anti-fungal activity, modulators of pathogenicity and activators of plant defense [38]. Another reported study of Žlabur et al. [26] investigated the bioactive components of chamomile extract, such as flavonoids, coumarins, phenolic acids, glucosides, sesquiterpenes and antioxidant vitamin, in addition to antioxidant activity with toxicity against *Vibrio fischeri* [28]. Other investigations pointed out that the exogenously applicable of biotic and abiotic agents could be affecting the physiological and metabolites of host plants, during activation of defense gene and generation of some antioxidant substances [46,49]. Likewise, the *Trichoderma* spp. (e.g., *T. hamatum*, *T. viride*, and *T. harzianum*) were showed to reduced significantly the pre-and post-emergence damping off disease of bean under artificial infection with pathogens [50], with additional significant increase in bean plant features and peroxidase and poly phenol oxidase activities compared to the control.

Commonly, the knowledge concerning the behavior of how *Trichoderma* spp. antagonism *P. ultimum*, is a vital for management of bean disease pathogen, whereby there are several mechanisms were suggested, i.e., production of lytic enzymes, antifungal antibiotics, competitors with pathogens and promotion of plant growth [51]. As well as, the potentiality of some *Trichoderma* spp against pathogenic fungi could be due to production of secondary metabolites such as, pyrones, koninginins, viridian, gliovirin, peptaibols and other negligible constituents [52].

- Photosynthesis pigments

Total chlorophyll, chlorophyll a, chlorophyll b and carotenoids were determined in Table 4. Generally, *P. ultimum*-infested soil decreased the total chlorophyll contents in kidney bean leaves significantly compared to the other pathogens. Furthermore, the mixture of extracts and *Trichoderma* showed superiority with significant values in total chlorophyll compared to the other treatments. Additionally, the carotenoid content of leaves showed a significant increase as a response to treatments compared to *Pythium* alone. These results follow those of reported investigations, which showed the role of *Trichoderma harzianum* in increasing the total chlorophyll content in potato, in addition to increasing antioxidant enzymes [47]. Moreover, *Pseudomonas aeruginosa* KMPCH and rhizobacteria showed a vital role in induction of systemic resistance in bean [10]. The *Trichoderma* spp. were also found to be effective against enormous soil borne fungi, especially their ability to induce plant resistance against foliar disease pathogen in bean, such as *Uromyces appendiculatus* [10], additionally, production of some antifungal agents, e.g., cellobiohydrolase, N-acetyl-β-glucosaminidase, trypsin like protease and β-glucosidase [53].

#### 2.2.4. Disease Symptoms of Bean (*Phaseolus vulgaris*, L.) as *Pythium* Pathogen Infection

**Figure 5:**
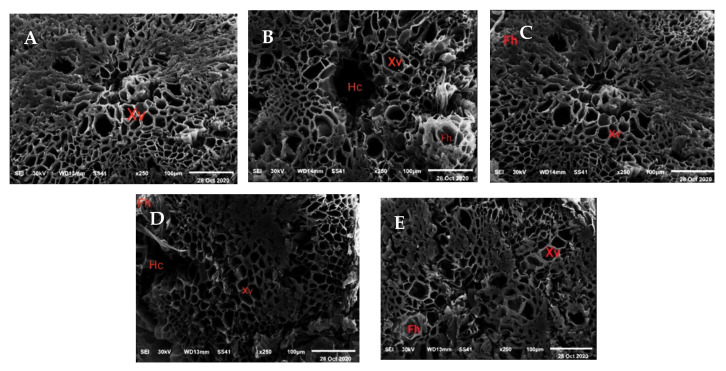
SEM micrograph of kidney bean root cross sections under infested soil with *Pythium ultimum*, (**A**): check (treated with fungicide), (**B**): fungal infection without treatment, (**C**): treated with extract + T. h., (**D**): treated with *Trichoderma harzianum* and (**E**): treated with extract. Xv = Xylem vessels, Hc = hydrolyzed cells, Fh = Fungal hyphae.

In infected soil, the disease symptoms appeared as necrotic lesions on vegetative growth, root rot, lower stem rot, wilt and subsequent plant death before the flower stage. Figure 5 illustrates the clear differences of kidney bean root structure that were found among cross sections under scanning electron microscope (SEM) of normal plant (Figure 5A) and plant infected with *P. ultimum*. The root section of an infected root (Figure 5B) showed remarkable differences occurring mainly in a cross-section shape containing the epidermis, cortex, vascular cylinder and pith cells. Complete destruction was reported in several areas of the epidermis, separation and hydrolysis and eventually maceration in some area of cortex tissue and degradation and dissolution of pith cell components leading eventually to cell death and presence of black areas in the central part of the root cross section. Fungal growth hyphae could be clearly seen in the inter-and intera-cellular spaces of cortex tissue. While, the dimensions of xylem vessels were increased in infected root due to the plant’s attempts at adaptating to compensate for the lack of water absorption. Otherwise, other treatments led to a decrease in the injurious effects of *P. ultimumon* root structure. The extract of chamomile flower was the most effective followed by the combination treatment of both *T. harzianum* and chamomile flower extract.

Fungal infection causes anatomical changes in different plant organs such as the changes in parenchymatous cell walls which involve swelling, less of fibrillary wall, hydrolysis or dissolution of cell components and eventually maceration of the tissues due to an increase in ethylene production [54]. Ethylene promoted the activity of exo-and endo cellular hydrolytic enzymes i.e., pectinethylestrase, polyglacturonase and cellulose [55]. Moreover, El-Hai, et al. [56] showed deformation in the anatomical structure in the basal portion of soybean stem infected with *M*. *phaseolina*, *R. solani*, *F. oxysporum* and *F. solani* which occurred mainly in the epidermis, cortex and pith. They observed complete disruption in the epidermal cells and sever plasmolysis in the cortical cells with destruction of the outer cortical cells. The infected kidney bean by mixture pathogenic fungi (*F. oxysporum*, *F. solani*, *R. solani* and *P. ultimum*) led to complete destruction of the root epidermis and separation in some area of cortex tissue followed by degradation and dissolution of cell components [57]. In our investigation, the increase in the dimensions of xylem vessels in root cross sections under infection of *P. ultimum* might be because of the plant’s attempts to compensate for the lack of water absorption which occurred due to the obstruction of some vessels by the fungal hyphae [58,59].

## 3. Materials and Methods

### 3.1. Preparation of Matricaria Chamomilla Flower Extract

Dry flowers of *Matricaria chamomilla* were obtained from the Agricultural Research Center (ARC, Giza, Egypt). Extraction process was prepared according to the method described by [60]. 100 g of *Matricaria chamomilla* whole dry flowers were extracted accurately using 1 L of deionized water heated to 60 °C for 90 min using a horizontal water bath shaker (Memmert WB14, Schwabach, Germany). Whatman no.1 filter paper (Whatman International Ltd., Kent, UK) was used to filter the extract. The filtrate was adjusted using deionized water in volumetric flasks to 500 mL and filtered through a Büchner funnel then stored at −18 °C for later use.

### 3.2. Fractionation and Identification of Phenolic Compounds

Phenolic compounds were identified using high performance liquid chromatography (HPLC) Technique at Food Technology Research Institute (FTRI), Agricultural Research Center according to Määttä, et al. [61]. This methodology was used for obtaining the chromatogram of each standard phenolic compound as well as a mixture of all the phenolic compounds. The standard phenolic compounds were purchased from reputed manufacturers such as Sigma-Aldrich (Cairo, Egypt). HPLC was conducted using a Hewlett-Packard instrument containing a 1100 series quaternary pump, diode array detector and an autosampler all linked to The Chemstation data handling system (Waldbronn Analytical Division, Waldbronn, Germany). Phenolic compounds were separated using LiChroCART Purospher RP-18e column (125 × 3 mm^2^ i.d., 5 µm, Merck, Darmstadt, Germany) with a guard column of the same material (4 × 4 mm^2^) used as protection. Finnigan MAT LCQ ion trap mass spectrometer (San Jose, CA, USA) with an attached Rheos 400 HPLC pump (Danderyd, Sweden) was used for Liquid chromatography–mass spectrometry (LC-MS) analysis. LC-MS is an analytical chemistry technique that combines the physical separation capabilities of liquid chromatography with the mass analysis capabilities of mass spectrometry. Conditions for the initial ionization in the positive and negative ionization modes included capillary voltages at +4.5 and −3 kV and a temperature at 225 °C. The MS data was acquired as full scan mass spectra at m/z 150-1500 by using 200 ms for collection of the ions in the trap. Tandem mass spectrometry or MS/MS is a technique in instrumental analysis where two mass analyzers are coupled together using an additional reaction step to increase their abilities to analyse chemical samples. MS/MS was performed using helium as the collision gas, and the collision energy was set at 30%. MS revealed the positive or negative molecular ions; MS/MS broke down the most abundant ones with dependent collision-induced dissociation.

The percentage peak area method uses the area of the target component peak as a proportion of the total area of all detected peaks to analyze quantity. This method is used to determine changes in concentration of a known sample mixture, or to determine an approximate concentration of a sample mixture. Retention time and peak area were used to calculate the concentrations of phenolic compounds content compared with standard calibrated polyphenols by analyzing the data of Hewlett Packard software.

### 3.3. Evaluation of Chamomile Flower Extracts Concentrations on Pythium Ultimum Growth

The response of linear growth of *Pythium ultimum* to aqueous extract of chamomile (*Matricaria chamomilla*, L.) flower was evaluated. From the extract, four concentrations (1.5, 2.0, 2.5 and 3.0%) were incorporated in potato dextrose broth media flasks by adding the appropriate amount of each concentration to the melted medium and then sterilized. Flasks without any addition were used as control. Disks (5 mm in diameter) taken from the growing edge of 5-day-old colony of *Pythium* were used singly inoculated the prepared flasks. The flasks were incubated at (25 °C). Three replicates were used per concentration. The fungal linear growth was calculated for three consecutive days from incubation.

### 3.4. Antifungal activity of Trichoderma Harzianum

- Dual Culture Assay

The potential of *T. harzianum* against *P. ultimum* was evaluated using a dual culture technique [62] by which the tested antagonist mycelial disc (5 mm) fungus was taken from 5-day-old culture which it paired against the same sized mycelial disc of pathogen fungus at the opposite end on 9 cm diameter of PDA Petri dishes. Both the pathogen and antagonist disc were inoculated at equal distances (1 cm) from the petri plate periphery. The PDA plates were incubated at 25 ± 2 °C. The growth of the pathogen and the control were recorded. The percent inhibition of radial growth was calculated using the following equation:

I = (R1 − R2)/R1 × 100(1)

where I = inhibition of radial growth. R1 = outward growth of the pathogen in control. R2 = radial growth of the pathogen in dual culture with antagonists.

Based on the previous screening, after five days of dual growth antagonism reaction of *T. harzianum* was evaluated by using a scale of five degrees, to detect growth and interaction between dual mycelia. The 1 to 5 scale rates the antagonism reaction, in which the first degree indicates that *Trichoderma* completely overgrowing pathogen and fifth degree is the opposite [63].

### 3.5. Evaluation of M. Chamomilla Flower Extract Concentration on T. harzianum Linear Growth

The effect of 2.0, 2.5 and 3%concentration of *M. chamomilla* extract was evaluated on *T. harzianum* linear growth by incorporating in potato dextrose broth media flasks and then sterilized. Flasks without any addition were used as control. Disks (5 mm in diameter) taken from the growing edge of 5-day-old colony of *T. harzianum* were used to singly inoculated the prepared flasks and incubated at (25 °C). Three replicates were used per concentration. The fungal linear growth was calculated for three consecutive days from incubation. *T. harzianum* was filtrated, fresh and dry weight of mat was recorded with three replicates.

### 3.6. Evaluation of Chamomile Flower Extract on T. harzianum Sporulation

To investigate the enhancing of chamomile flower extract on *T*. *harzianum*, the fungus was gently scraped from the Petri-dish with a sharp spatula and washed several times with a total volume of 50 mL of sterilized water. The number of spores per mL was determined using a hemocytometer compared to the control.

### 3.7. Greenhouse Experiment

The effect of *M. chamomilla* flower extract and *T. harzianum* either each of them separately or in combination between them was evaluated against *Pythium* pathogen under greenhouse conditions.

#### 3.7.1. Inoculum Preparation

- The inoculum of *Pythium*

*P. ultimum* inoculum was prepared by growing on potato dextrose agar plates and incubated at 25 °C for five days and then mycelial plugs were carried on sterilizing medium of sorghum: coarse sand: water (2:1:2 *v*/*v*) and incubated at 25 °C for ten days; to be ready to use.

- *T. harzianum* inoculum

*T. harzianum* inoculum was prepared using 14 days old culture grown on potato dextrose broth under static conditions (25 ± 2) as active ingredients.

#### 3.7.2. Greenhouse Evaluation of Chamomile Flower Extract and/or *T. harzianum* on *Phaseolus vulgaris* Pathogen

Pots were filled with 8 kg/pot antiseptic soil; clay: sand (2:1, *v*/*v*) and singly infested with the previous prepared pathogen inoculum at the rate of 0.4% (*w*/*w*), with irrigate regularly with tap water and left for one week to warranty the fungus spread. On the day of planting, the beans were soaked in 2.5% of *M. chamomilla* concentration and *T. harzianum* filtrate either each of them separately or in combination between them according to the treatment. Additionally, chemical fungicide (F) used was (56% Sc) Dose that recommended by the Ministry of Agricultural, was 250 cm/100 L.

The treatments applied were; (1) chamomile extract (2) *T. harzianum* (3) chamomile extract + *T. harzianum*, (4) Fungicide, (5) *Pythium* only, (6) chamomile extract + *Pythium*, (7) chamomile extract + *T. harzianum*+ *Pythium* and (8) *T. harzianum*+ *Pythium*. All pots were organized in randomized block design and kept in the greenhouse.

- Disease assessment

After ten days, the percentage of rotted beans (un-emerged beans), post infected seedlings (percentage of dead seedlings after 30 days from planting) and plant survival was recorded.

- Vegetative growth parameters

Five plants of each treatment were carefully harvested after five weeks from planting, rinsing with tap water to remove any soil particles and the following parameters were recorded, shoot, root and plant length (cm), root fresh and dry weight (g), plant fresh and dry weight (g) and leaf area.

- Chlorophylls and carotenoids content of investigated leaves

The totals of the following, chlorophyll a, b and carotenoids were evaluated according to Wellburn [64]. Fresh leaves (0.05 g) were placed inside a test tube, softened and soaked in 10 mL methanol at 4 °C overnight in the presence of trace amounts of sodium bicarbonate to inhibit the function of chlorophyllase enzymes, the test tube is then sealed using aluminum foil to prevent photooxidation from occurring. The chlorophyll contents were then measured through spectrophotometry at 452.5, 650 and 665 nm respectively using the following calculations [65]:

Total Chlorophyll = (25.5 × E650) + (4 × E665)(2)

Chlorophyll a = (16.5 × E665) − (8.3 × E650)(3)

Chlorophyll b = (33.8 × E650) − (12.5 × E665)(4)

Carotene = (4.2 × E452.5) − (0.0264 × Chlorophyll a) − (0.496 × Chlorophyll b)(5)

(6) $$\begin{array}{c} \mathrm{Chlorophyll}\;\mathrm{or}\;\mathrm{carotene}\;\left(\mathrm{mg}/g\;\mathrm{fresh}\;\mathrm{weight}\right)\;\\ \;\;\;\;\;\;=\frac{\mathrm{Chlorophyll}\;\mathrm{or}\;\mathrm{carotene}\;\mathrm{content}\;\times \;\mathrm{volume}\;\mathrm{of}\;\mathrm{methanol}}{1000\;\times \;\mathrm{weight}\;\mathrm{of}\;\mathrm{sample}\;\left(g\right)} \end{array}$$

- Total polyphenols content

To measure the total phenolic content, 0.1 g of air-dried leaves were dissolved in 1 mL of distilled water of which 0.1 mL was taken and added to a solution of exactly 2.8 mL of distilled water, 2.0 mL of 2% (*w/v*) sodium carbonate and finally 0.1 mL of 50% (*v/v*) of Folin–Ciocalteu reagent utilizing the Folin–Ciocalteu reagent method [66]. The mixture was then incubated at room temperature for 30 min before the solution was spectrophotometrically (Spekol 11 spectrophotometer, Analytik Jena AG, Jena, Germany) measured at 750 nm with distilled water used as a blank. For quantitative assessment a standard curve of gallic acid (0–200mg/L) was prepared in the same manner. Total phenol contents were expressed as milligram gallic acid equivalent (GAE)/g based on dry weight.

- Total flavonoids content

To measure the total flavonoids colorimetrically of the air-dried leaves, 0.1 g of the leaves were dissolved in 1 mL of distilled water of which 0.5 mL was then taken and 1.5 mL of 95% ethyl alcohol, 0.1 mL of 10% aluminum chloride (AlCl_3_), 0.1 mL of 1 M potassium acetate (CH_3_COOK) and 2.8 mL of distilled water was added [67]. The resulting solution was then incubated for 40 m at standard room temperature before the solution was measured using a spectrophotometer at 415 nm with distilled water used as a blank. A standard curve was constructed using quercetin as the standard for flavonoids (0–50 mg/l). The total concentration of the flavonoids contents were measured as milligram quercetin equivalent (QE)/g based on dry weight.

- Determination of antioxidant activity by the DPPH and ABTS radicals scavenging methods

The free radical scavenging activity was determined according to [68] using different concentrations of 2,2-diphenyl-1-picrylhydrazyl (DPPH) by measuring the bleaching of the purple color of DPPH, the absorbance was measured at 517 nm and the percentage of inhibition was calculated. The ABTS (2,2’-azino-bis (3-ethyl benzothiazoline-6-sulfonic acid) assay was based on the method of Christodouleas, et al. [68]. The absorbance of the resulting greenish-blue solution was recorded at wavelength 734 nm, the decrease in the absorbance is expressed as a percentage of inhibition which was calculated.

- Polyphenol oxidase and peroxidase activities

Extraction and activity of both enzymes were determined using a spectrophotometric method based on an initial rate of increase in absorbance at 410nm were carried out at 4 °C, according to Seleim, et al. [69].

### 3.8. SEM Analysis

The anatomical changes in bean root due to the pathogen and other treatments were studied by taking cross sections, gold-coating them and examining at various magnifications using SEM (TEM-2100, JEOL, Tokyo, Japan) attached to an accelerating voltage of 200 kV at the Central Laboratory, Electron Microscope Unit, Mansoura University, Egypt [70].

### 3.9. Statistical Analysis

The statistical analysis software CoStat version 6.4 (CoHort Software, Pacific Grove, CA, USA) was used to perform the analysis of variance of the data. Duncan’s new multiple range test at probability (*p*) level ≤ 0.05 was applied. Some of the experimental data were presented as means ± standard deviation (±SD) and at least six replicates were used.

## 4. Conclusions

Our investigative study aimed to investigate the effect of chamomile (*Matricaria chamomilla*, L.) flower extract and *T. harzianum* either alone or combined as bioagents against *P. ultimum* with an effect up to 30–81.6% radial growth reduction in both of in vitro and in vivo applications. The potentiality of both chamomile extract and *T. harzianum* against *P. ultimum* was obtained. Furthermore, the fractionation of *M. chamomilla* extract was performed to investigate its active compounds which showed several polyphenolic compounds. The in vivo response of percentages of root rot, seedling rot and survival for both chamomile flower extract and *T. harzianum* was determined to be up by 64%. The biochemical parameters such as chlorophyll, carotenoids, and antioxidant enzymes increased with use of the therapeutic treatments and the cross sections of roots of bean were investigated using SEM with a decrease in symptomatic features of diseases as a response of therapeutic treatments. In conclusion, the application of both *T. harzianum* and/or *M. chamomilla* extracts in the control of bean *Pythium* pathogen showed significant results.

## Figures and Tables

**Table 1:** Fractionation and identification of phenolic compounds of *Matricaria chamomilla* flowers extract using HPLC technique.

Compound/Structure	Retention Time (min)	Concentration (ppm)	Compound/Structure	Retention Time (min)	Concentration (ppm)
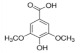 Syringic acid	3.431	137.446	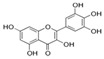 Myricetin	12.850	1587.823
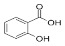 Salicylic acid	3.733	66.672	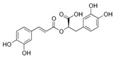 Rosmarinic acid	13.624	370.598
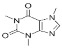 Caffeine	6.215	79.975	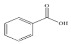 Benzoic acid	13.797	414.887
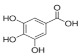 Gallic acid	6.813	77.146	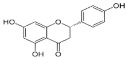 Naringenin	13.977	400.997
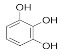 Pyrogallol	7.245	324.612	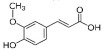 Ferulic acid	15.243	47.982
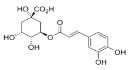 Chlorogenic acid	8.493	38.02	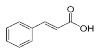 Cinnamic acid	15.410	9.992
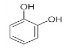 Catechol	9.621	11.289	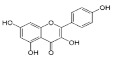 Kaempferol	16.766	118.772
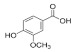 Vanillic acid	10.312	112.577	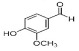 Vanillin	19.026	17.298
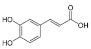 Caffeic acid	10.483	99.691	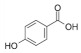 *p*-Hydroxybenzoic acid	20.451	30.563
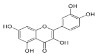 Quercetin	11.305	927.727	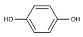 Hydroquinone	20.779	20.133
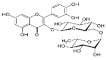 Rutin	11.569	127.074	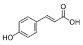 *p*-Coumaric acid	22.467	23.591
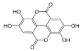 Ellagic acid	12.507	17.016	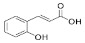 *o*-Coumaric acid	24.345	20.999

**Table 2:** The percentage of rotted seeds and infected seedlings as well as survival plants of *Phaseolus vulgaris* as response to different treatments.

Treatments	Rotted Seeds	Infected Seedling	Survival%
*M*	30 b	12 a	58 bc
*T. harzianum*	38 b	8 ab	54 bc
*T + M*	28 b	8 ab	64 b
Fungcide	12 c	4 b	84 a
*Pythium*	56 a	14 a	30 d
*Ex + Pythium*	32 b	12 a	64 b
*T + M + Pythium*	38 b	12 a	50 bc
*T + Pythium*	40 b	16 a	44 cd

Different letters within each column means values are significantly different at *p* ≤ 0.05.

**Table 3:** Biochemical features of *Phaseolus vulgaris* plants as response of different treatments.

Treatment	Total Polyphenols	Total Flavonoids	Polyphenol Oxidase	Peroxidase	ABTS Inhibition%	DPPH Inhibition%
*M. chamomilla*	28.699 d	15.056 d	9.776 d	0.619 d	29.936 d	13.677 e
*T. harzianum*	27.755 d	14.189 d	9.414 d	0.599 d	21.726 e	11.495 f
*T + M*	43.222 a	28.469 a	15.353 a	0.948 a	58.234 a	26.151 a
Fungcide	40.960 b	26.381 b	11.152 c	0.897 b	55.647 b	25.036 b
*Pythium*	26.071 e	12.632 e	8.766 e	0.561 e	14.129 g	7.147 h
*M + Pythium*	32.945 c	18.980 c	11.408 c	0.716 c	33.658 c	17.919 d
*T + M + Pythium*	40.079 b	25.575 b	14.150 b	0.877 b	34.495 c	19.557 c

Different letters within each column means values are significantly different at *p* ≤ 0.05.

**Table 4:** Chlorophyll A, chlorophyll B and total chlorophyll of *Phaseolus vulgaris* plants as responded to different treatments.

Treatment	Chlorophyll A	Chlorophyll B	Total Chlorophyll	Carotenoids
*M. chamomilla*	2.814 a	1.651 b	4.466 b	0.679 c
*T. harzianum*	2.123 b	1.418 c	3.542 d	0.578 d
*T + M*	2.908 a	1.875 a	4.784 a	0.934 a
Fungcide	1.592 d	1.180 e	2.771 f	0.529 e
*Pythium*	1.526 d	1.113 e	2.639 f	0.286 f
*M + Pythium*	2.136 b	1.430 c	3.566 d	0.894 b
*T + M + Pythium*	2.248 b	1.598 b	3.846 c	0.597 d

Different letters within each column means values are significantly different at *p* ≤ 0.05.

## Data Availability

See MDPI Research Data Policies at https://www.mdpi.com/ethics.

## Sample Availability

Samples of the compounds are not available from the authors.
